# A rare surgical emergency during pregnancy: a case report

**DOI:** 10.1093/jscr/rjaf1080

**Published:** 2026-01-19

**Authors:** Zouhry Ibrahim, Guetmy Zakaria, Alaoui Babana Amina, Mohammed Boufeloussen, Khedid Yahia, Alami Faricha Hassan

**Affiliations:** Visceral Surgery, National Institute of Oncology, Rabat, Morocco; Department of Visceral Surgical Emergencies Rabat, Mohammed V Souissi University, Rabat-Sale-Zemmour-Zaer, Morocco; Department of Visceral Surgical Emergencies Rabat, Mohammed V Souissi University, Rabat-Sale-Zemmour-Zaer, Morocco; Department of Visceral Surgical Emergencies Rabat, Mohammed V Souissi University, Rabat-Sale-Zemmour-Zaer, Morocco; Department of Visceral Surgical Emergencies Rabat, Mohammed V Souissi University, Rabat-Sale-Zemmour-Zaer, Morocco; Department of Visceral Surgical Emergencies Rabat, Mohammed V Souissi University, Rabat-Sale-Zemmour-Zaer, Morocco

**Keywords:** pregnancy, adnexal torsion, adnexectomy

## Abstract

Acute pelvic pain during pregnancy often presents a diagnostic challenge. We report a case of adnexal torsion occurring in the third trimester of pregnancy in order to highlight this diagnosis, for which only early management can prevent irreversible ischemic damage that may compromise future fertility. The patient was a nulliparous woman at 30 weeks of amenorrhea who initially presented with symptoms suggestive of appendicitis. Through a McBurney incision, surgical exploration revealed a necrotic right ovary associated with reactive appendicitis. An oophorectomy and appendectomy were performed. The postoperative course was uneventful.

## Introduction

Adnexal torsion is a rare condition resulting from the total or partial rotation of the adnexa around its vascular axis [1]. It may be facilitated by the presence of an adnexal mass or, more rarely, occur on normal adnexa [2]. The importance of this condition lies in its diagnostic difficulty, due to the upward displacement of the ovary in advanced pregnancies, which can mimic other surgical emergencies such as acute appendicitis, as well as in the choice of appropriate therapeutic management [3]. We report a case of adnexal torsion involving a normal ovary that occurred during the third trimester of pregnancy in a young nulliparous woman.

## Case presentation

**Figure 1:**
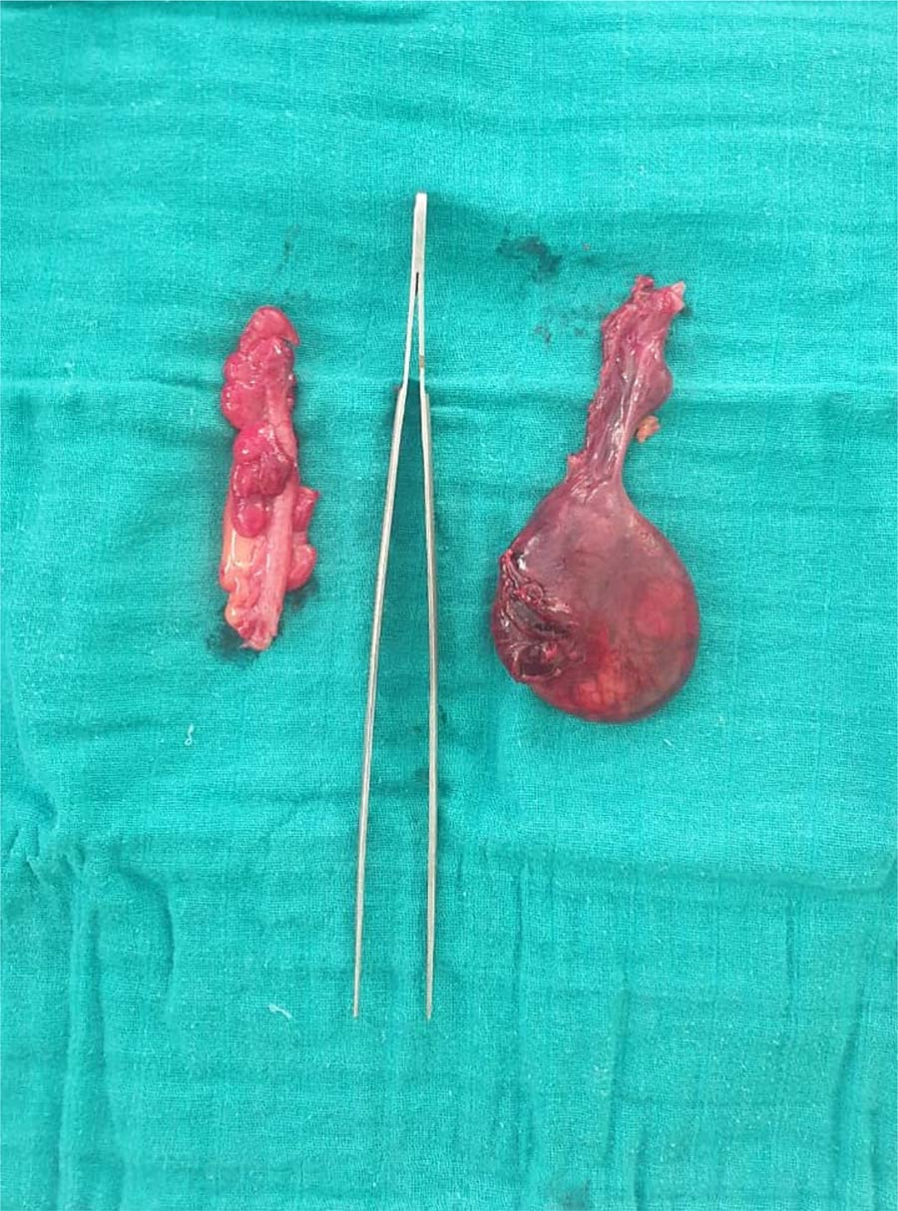
Image showing the two surgical specimens: the appendix on the left and the adnexa on the right.

A 25-year-old nulligravid patient, with no significant medical history, presented with right iliac fossa pain evolving over two days, at 30 weeks of amenorrhea. Clinical examination revealed a conscious, afebrile patient in fairly good general condition. The abdomen was soft with tenderness in the right iliac fossa. Gynecological examination showed a relaxed uterus; on speculum examination, the cervix appeared macroscopically normal, with no metrorrhagia or leukorrhea. On vaginal examination, the cervix was long, closed, and posterior. Abdominal ultrasound revealed a single viable intrauterine pregnancy with a homogenous placenta normally inserted and a normal amount of amniotic fluid. The appendix appeared enlarged with a 20 × 30 mm collection suggestive of appendicitis complicated by an abscess, while the right ovary was not visualized. Laboratory investigations showed elevated C-reactive protein and leukocytosis. A diagnosis of acute appendicitis was made. The patient underwent surgery under general anesthesia through a McBurney incision. Intraoperative findings revealed a necrotic, twisted right ovary associated with a reactive inflammatory appendix (Fig. 1). A right oophorectomy and appendectomy were performed. Postoperatively, the patient received uterine relaxant therapy. The postoperative course was uneventful, and the prognosis for a term vaginal delivery was favorable.

## Discussion

Adnexal torsion during pregnancy is a rare surgical emergency. Its incidence ranges from 3 to 5 per 10 000 pregnancies. Between 8 and 28% of torsion cases occur during pregnancy, most frequently in the first trimester, but it can be diagnosed at any gestational age [3, 4]. It affects women of all ages, with an average age of onset around 30 years [5]. Typically, torsion occurs on a pathologic ovary. The most common risk factors include the presence of an ovarian cyst, a history of pelvic surgery, or assisted reproductive technology. However, it can also occur on a normal ovary [5, 6]. Diagnosis during pregnancy is complex, as it requires ruling out both classical differential diagnoses and those specific to pregnancy (such as miscarriage, retroplacental hematoma, or uterine rupture). Furthermore, both clinical examination and imaging become more challenging due to uterine enlargement and the concomitant upward displacement of the ovary into the abdominal cavity. The clinical presentation is dominated by acute, intense lower abdominal pain in the iliac fossa, sometimes preceded by recurrent, transient episodes of sub-torsion. Nausea, vomiting, and low-grade fever are frequently associated symptoms. On examination, abdominal guarding may be present, and vaginal examination may reveal a painful cul-de-sac, though this may be difficult to assess [6, 7]. Ultrasound is the gold standard imaging modality. It helps to exclude differential diagnoses, identify predisposing factors, and detect indirect signs of ischemia. The initial interruption of venous flow leads to reactive edema. Additionally, an increased number of cortical follicles is a nonspecific feature that has often been described in cases of torsion involving a normal ovary. The usefulness of Doppler assessment of ovarian vessels remains controversial: while the absence of a Doppler signal confirms the lack of arterial or venous flow and thus supports the diagnosis of torsion, a normal Doppler signal does not exclude it. Magnetic resonance imaging (MRI) is a valuable complementary imaging technique during pregnancy, offering similar diagnostic accuracy to ultrasound with greater precision. The combination of Doppler ultrasound and MRI can be helpful but should not delay surgical management [5, 7]. Adnexal torsion constitutes a true surgical emergency. Currently, laparoscopy is recommended for pregnancies with a gestational age under 17 weeks of amenorrhea, provided certain safety measures are respected: the use of an open-entry technique, insufflation pressure maintained between 8 and 12 mmHg, appropriate trocar positioning, and gentle uterine manipulation. The decision between conservative and radical treatment depends on the appearance of the adnexa 10 minutes after detorsion. Conservative management is preferred when viable tissue remains, allowing functional recovery in approximately 90% of cases. In advanced stages with necrotic, black, friable adnexa showing no recovery after detorsion, adnexectomy is advisable. However, some authors advocate a conservative approach even in cases of questionable viability, given the high regenerative capacity of ovarian tissue. Ovarian pexy is essential in cases of ligamentous malformation or immediate recurrence of torsion [1].

## Conclusion

The diagnosis of adnexal torsion remains difficult, particularly during pregnancy. The symptomatology is poorly specific, and paraclinical investigations contribute little to establishing a positive diagnosis, though they remain important for excluding various differential diagnoses and for identifying an adnexal pathology. The surgical procedure should be as conservative as possible and consists of adnexal detorsion; oophoropexy should not be performed systematically. The pregnancy outcome is generally favorable.
